# Topical Application of Ketoprofen Improves Gait Disturbance in Rat Models of Acute Inflammation

**DOI:** 10.1155/2013/540231

**Published:** 2013-08-07

**Authors:** Yosuke Amagai, Akane Tanaka, Akira Matsuda, Kumiko Oida, Kyungsook Jung, Sho Nishikawa, Hyosun Jang, Saori Ishizaka, Hiroshi Matsuda

**Affiliations:** ^1^Cooperative Major in Advanced Health Science, Graduate School of Bio-Applications and System Engineering, Tokyo University of Agriculture and Technology, 3-8-5 Saiwai-cho, Fuchu, Tokyo, Japan; ^2^Laboratories of Comparative Animal Medicine, Tokyo University of Agriculture and Technology, 3-8-5 Saiwai-cho, Fuchu, Tokyo, Japan; ^3^Veterinary Molecular Pathology and Therapeutics, Division of Animal Life Science, Tokyo University of Agriculture and Technology, 3-8-5 Saiwai-cho, Fuchu, Tokyo, Japan

## Abstract

Arthritis is a disabling health problem and commonly develops in the late stages of life; the condition is typically accompanied by chronic pain. For the assessment of pain severity and therapeutic effects of analgesic drugs, we recently developed a gait analysis system, which provides an index of pain severity based on walking stride disturbance. Using this system, we evaluated the therapeutic effect of topical nonsteroidal anti-inflammatory drugs (NSAIDs) in rat models of acute inflammation. We found that the gait analysis system is more sensitive than conventional evaluation methods, such as measurement of swelling or analgesia, which indicated the superiority of our system for drug screening. The approach also indicated that ketoprofen is superior to other NSAIDs for providing pain relief because of its higher skin permeability. To the best of our knowledge, this is the first report demonstrating the effectiveness of topical NSAIDs in experimental animal models of acute inflammation.

## 1. Introduction

Osteoarthritis (OA) is the most common form of arthritis and the leading cause of chronic disability [1]. Rheumatoid arthritis (RA) is a progressive autoimmune disease characterized by chronic inflammation in the limbs and joints and affects 1% of the adult population [2]. Since chronic pain caused by OA and RA negatively affects the patients' quality of life, nonsteroidal anti-inflammatory drugs (NSAIDs) are routinely prescribed for pain relief. The use of topical NSAIDs is not new in the treatment of acute and chronic pain and has been shown to provide adequate pain relief with many important advantages. These advantages include protection of the active compound from gastric enzymes, avoidance of the hepatic first-pass effect, and reduction in the risk of gastrointestinal adverse effects such as ulcer, bleeding, and perforation. Furthermore, several clinical trials in OA patients have indicated that compared to oral forms, topical formulations of NSAIDs have comparable therapeutic effects and a low incidence of adverse effects [3, 4].

Commonly used methods for assessing the severity of arthritis and the therapeutic effects of drugs in animals are based on the degree of inflammatory responses such as pain, edema, and analgesia in the paws and/or joints, which is subjectively determined by the investigator. The threshold for limb withdrawal or vocalization is a common parameter used to indicate pain-related behavior. However, patients with arthritis do not necessarily complain about stimulus-evoked pain, but rather about pain at rest or movement-induced pain [5, 6]. Although few methods are currently available to objectively evaluate spontaneous pain related to arthritis, gait analysis has been proven to be an objective and sensitive technique for detecting gait abnormalities in connection with pain in several arthritis models [7–9]. In previously reported studies, both velocity and stride length significantly decreased in arthritis models, which seems reasonable because patients with OA or RA often show from decreased joint utilization in association with pain, for example, when walking [10, 11]. It has been reported that systemic administration of drugs, including NSAIDs, can improve gait disturbance in rat models of arthritis. However, as far as we know, the therapeutic effects of topical NSAID formulations have not been studied in animal models.

In this study, the clinical benefit of topical NSAIDs for pain relief in carrageenan-induced acute inflammation was assessed using our recently developed gait analysis method [12, 13].

## 2. Materials and Methods

### 2.1. Drugs and Chemicals

The following products were evaluated in this study: patch formulations including a ketoprofen patch (20 mg/70 cm^2^; Keplat, Hisamitsu UK Ltd, London, UK), a diclofenac patch (180 mg/140 cm^2^; Flector, Bayer S.p.A, Milano, Italy), and a loxoprofen patch (50 mg/70 cm^2^; Loxonin tape, Daiichi Sankyo Co. Ltd., Tokyo, Japan) and gel formulations including a ketoprofen gel (2.5% w/w; Ketum, A. Menarini Pharmaceutical Ltd, Florence, Italy.), a diclofenac gel (1.16% w/w; Voltarol Pain-eze emulgel, Novartis Consumer Health, Inc., Horsham, UK), and a loxoprofen gel (1% w/w; Loxonin gel, Daiichi Sankyo Co Ltd, Tokyo, Japan). Other chemicals were obtained from commercial sources.

### 2.2. Animals

Male HWY rats aged 8 weeks (Japan SLC, Inc., Shizuoka, Japan, for gait analysis and histological analysis), and male Sprague-Dawley (SD) rats aged 5 weeks (Japan SLC, Inc., for the *γ*-carrageenan-induced edema model, and Clea Japan, Inc., Tokyo, Japan, for the yeast-induced hyperalgesia model) were used. All animals were kept in an air-conditioned room controlled for temperature (22 ± 3°C) and humidity (50% ± 20%) under a light/dark cycle (light on from 8 : 30 to 20 : 30). The animals were allowed food and water ad libitum except during the experiments. All animal experiments were performed with local committee approval in accordance with Laboratory Animal Welfare guidelines.

### 2.3. Measurement of Gait Disturbance

Rats were habituated to the experimental apparatus, including an acrylic wheel that revolved 4.0–6.0 rpm/min and trained to keep walking on it for 2 min in a day, 3 times in a week prior to experiments. On the day of the experiment, each rat was anesthetized with ether and a volume of 0.05 mL of 0.5% *γ*-carrageenan-saline solution was injected into the right knee-joint space. Walking behavior was recorded for 2 min using a DigiOn TVR (DigiOn Inc., Fukuoka, Japan). For the evaluation of therapeutic effects, each NSAID formulation was applied (patch: 20 cm^2^/paw, gel: 250 mg/paw) to the knee joint 4 h prior to (before administration) and/or 19 h after (after administration) *γ*-carrageenan injection for 4 h. For the full duration of drug administration, the rats were suspended with a rodent sling cover (Lomir Biomedical Inc., Canada) to keep each drug from moving from the site of application and to reduce opportunity for oral ingestion.

Gait analysis was conducted using GAIT software (Noveltec Inc., Kobe, Japan) [12, 13]. The software automatically calculates the swing time of both hind limbs in each step cycle, and the swing time index (STI) was determined using the following formula: swing time ratio (STR) = (swing time of normal hind limb)/(swing time of inflamed hind limb). When STR is greater than 1, STI = −[(1/STR) − 1], and when STR is less than 1, STI = STR − 1. As a single hind limb bears the weight during a swing, the swing time of one hind limb shortens if pain is experienced in the opposite hind limb, leading to a decrease in STI. The STI of each rat was calculated and averaged for 2 min.

### 2.4. Measurements of Hind Paw Edema and Pain Thresholds

Edema and hyperalgesia were induced by subcutaneous injection of 1.0% *γ*-carrageenan-saline solution and 20% yeast suspension, respectively, in the right hind paw [14, 15]. Each drug (patch: 7 cm^2^/paw, gel: 100 mg/paw) was topically applied to the right hind paw for 3 h prior to induction of inflammation. The right hind paw volume and pain thresholds were measured with a plethysmometer (MK-101CMP; MUROMACHI KIKAI Co., ltd., Tokyo, Japan) and by the randall-Selitto test with analgesy-meter (MK-201D, MUROMACHI KIKAI Co., ltd.), respectively, before drug administration and 3 h after the induction of inflammation. In some experiments, each drug (30 mg/0.1 mL/paw, dissolved in PBS) was mixed with yeast and directly injected into the right hind paw.

### 2.5. Histological Analysis

Histology of the knee joint was assessed in each of 3 control or NSAID-treated (with patch formulation of ketoprofen, diclofenac, or loxoprofen) rats. Each NSAID was applied for 4 h before the induction of arthritis with 0.5% *γ*-carrageenan-saline solution as described above. Twenty-four h later, the knee joints were removed, fixed in formalin, decalcified in 10% formic acid, embedded in paraffin, cut into 3 *μ*m sections, and stained with hematoxylin-eosin. The level of inflammatory cell inflammation was scored by a pathologist blinded to treatment conditions; the pathologist assessed the infiltration of neutrophils and mononuclear cells into the dorsal synovial membrane of the knee joint (0: no change, 1: mild, 2: moderate, and 3: severe).

### 2.6. *In Vitro* Skin Permeation Test

The cutaneous permeation profiles of NSAIDs were evaluated using hairless mouse skin and a Franz diffusion cell system. The dorsal skin was harvested from mice (Crl:SKH1-Hr^hr^ mice, Charles River, Yokohama, Japan) and the subcutaneous fat was removed. A piece of skin was mounted on a Franz diffusion cell, after which each NSAID solution (50 mg/mL, dissolved in acetone) was applied to the skin (15 *μ*L/4.9 cm^2^). Receptor fluid (PBS, pH 7.4, 10 mL) was collected every 2 h for 24 h. The surface temperature of the skin was maintained at 32°C. A 500 *μ*L aliquot of each receptor fluid specimen was mixed with 500 *μ*L of acetonitrile. After centrifugal filtration, the supernatant was used to determine the concentration of the test drug by liquid chromatography (Waters 2690, Waters Corp., Milford, MA, USA).

### 2.7. Statistical Analysis

The data are represented as mean ± SE. Statistical analysis was conducted using SAS Version 6.12 (SAS Institute Japan Ltd., Tokyo, Japan). One-way ANOVA and Bartlett test, followed by the Dunnett test, were used to compare mean values among the groups. The level of significance was set at *P* < 0.05.

## 3. Results

**Figure 1:**
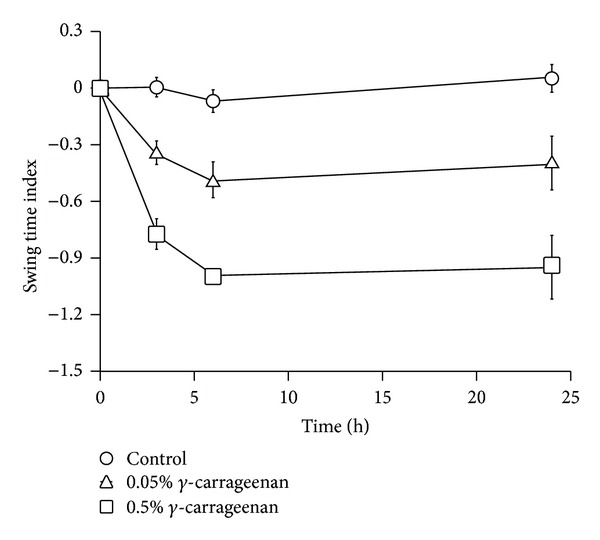
Validation of carrageenan-induced arthritis in an acute inflammation model. *γ*-carrageenan was injected into the hind paw, and gait disturbance was measured at 3, 6, and 24 h after injection. Each graph represents the mean ± SE of 4 animals.

**Figure 2:**
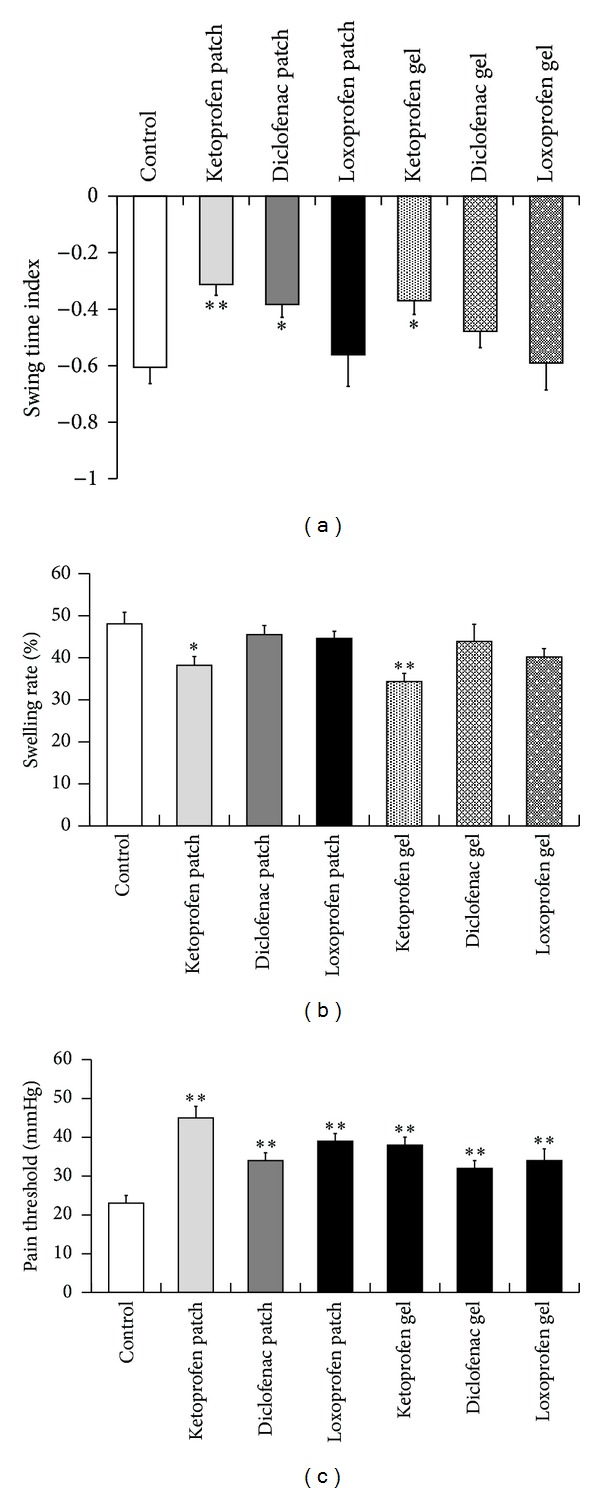
Comparison of the therapeutic effects of topical NSAID formulations in an acute inflammation model. Effects of topical NSAID formulations on gait disturbance (a) and hind paw edema (b) in a *γ*-carrageenan-treated inflammation model. Each column represents the mean ± SE of 6–18 animals. Asterisks show a significant difference from control, *P < 0.05, **P < 0.01. Hyperalgesia in yeast-treated inflammation model (c). Each column represents the mean ± SE of 10–20 animals. Asterisks show a significant difference from control, *P < 0.05, **P < 0.01.

**Figure 3:**
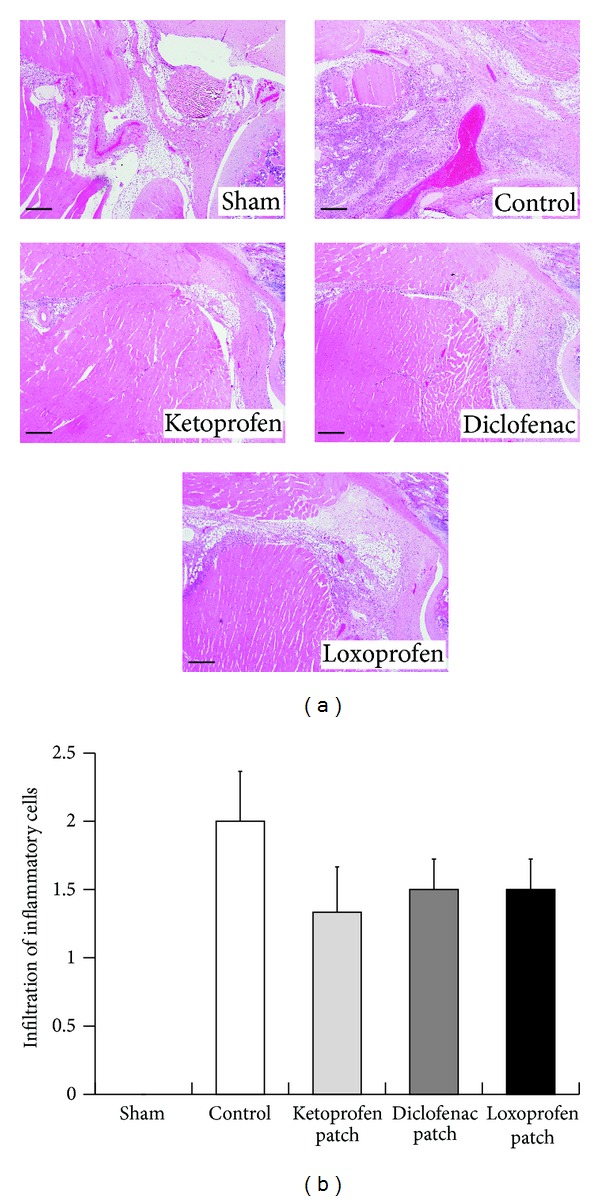
Histological analysis of knee joints of rats with *γ*-carrageenan-induced arthritis. Representative sections stained with hematoxylin and eosin (a). Scale bars are 300 *μ*m. Histopathological scores for infiltration of neutrophils and mononuclear cells (0, no change; 1, mild; 2, moderate; and 3, severe) (b). Each column represents the mean ± SE of 3 animals.

First, alterations of gait were assessed in the carrageenan-induced acute inflammation model. Carrageenan injection into the plantar surface of the hind paws decreased the STI in a dose-dependent manner, reaching the lower peak at 6 h, which lasted for at least 18 h (Figure 1). Using this model, we evaluated the therapeutic effect of topical NSAIDs on walking performance. Each NSAID (ketoprofen, diclofenac, or loxoprofen), in the form of a gel or patch, was topically applied to the skin around the hind knee-joint before *γ*-carrageenan injection. Loxoprofen application did not show any improvement in gait, while ketoprofen dramatically improved gait disturbance (Figure 2(a)). In contrast, diclofenac slightly improved the STI, but only when the patch form was applied (Figure 2(a)). In addition to gait assessment, we also measured edema. However, a reduction in the swelling rate was barely detectable in the ketoprofen treatment group (Figure 2(b)). Furthermore, histological analysis showed that the infiltration of inflammatory cells was suppressed in the ketoprofen patch/gel treatment group, although it was not significantly different from that observed in the loxoprofen or diclofenac treatment group (Figures 3(a) and 3(b)). Hyperalgesia is another major parameter used to determine the efficacy of analgesic drugs [16]. To assess the effect of topical NSAIDs, pain threshold modulation by each agent was measured in a yeast-induced inflammation model. Pain threshold increased in all NSAID treatment groups, even in the loxoprofen treatment group that showed no STI improvement in the carrageenan model (Figure 2(c)).

**Figure 4:**
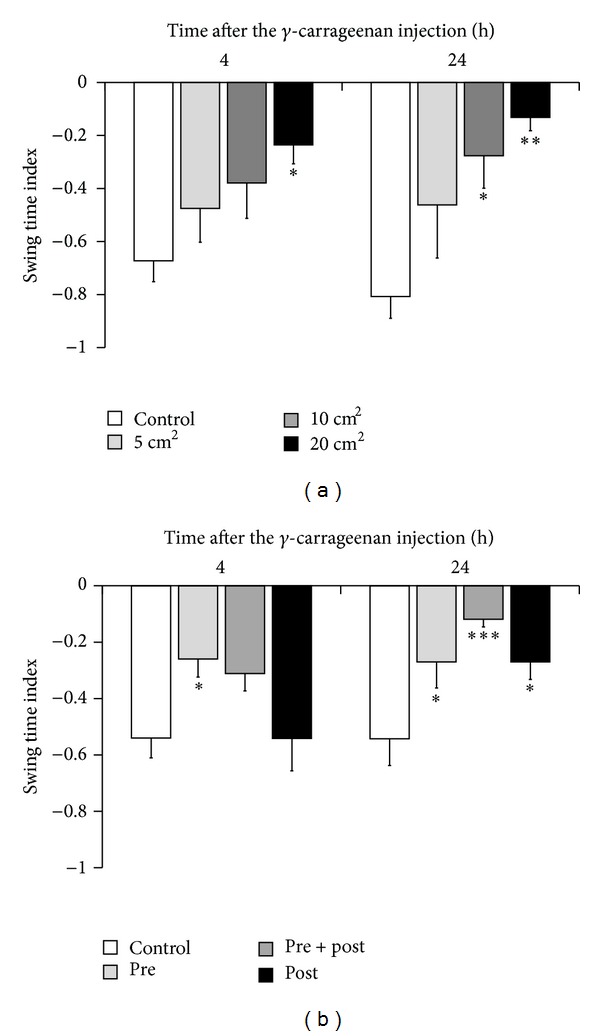
Effects of a ketoprofen patch on gait disturbance in *γ*-carrageenan-induced arthritis in rats. Evaluation of the timing of administration (a). The ketoprofen patch was applied on the affected knee joint before (pre), before and after (pre + post), or after (post) the induction of arthritis. Walking was evaluated with a GAIT system at 4 and 24 h after the injection of *γ*-carrageenan. A mock patch was used as a control. Each column represents the mean ± SE of 13-14 animals. *P < 0.05, ***P < 0.001, compared to STI in controls. Dose-dependent effects of the ketoprofen patch (b). A mock patch was used as a control. Each column represents the mean ± SE of 7 animals. *P < 0.05, **P < 0.01, when compared to STI in controls.

These results indicate that STI is the most sensitive parameter for the assessment of topical NSAID efficacy, at least with respect to these experimental models. To further examine whether gait analysis can provide quantitative measurements of therapeutic efficacy, STI changes were measured with various doses and durations of ketoprofen patch treatments involving application of several different sizes of ketoprofen patches; then, the therapeutic outcomes were compared. As shown in Figure 4(a), gait disturbance improved in a dose-dependent manner. We also compared the pretreatment or posttreatment potency, or a combination of both. Posttreatment application improved the STI 24 h after carrageenan injection, as shown in Figure 2(a). However, STI was not improved when measured 4 h after carrageenan injection. In contrast, treatment with ketoprofen before carrageenan injection showed superior therapeutic effects compared to posttreatment ketoprofen application (Figure 4(b)). The combination treatment synergistically improved STI, especially at 24 h after carrageenan injection (Figure 4(b)).

**Figure 5:**
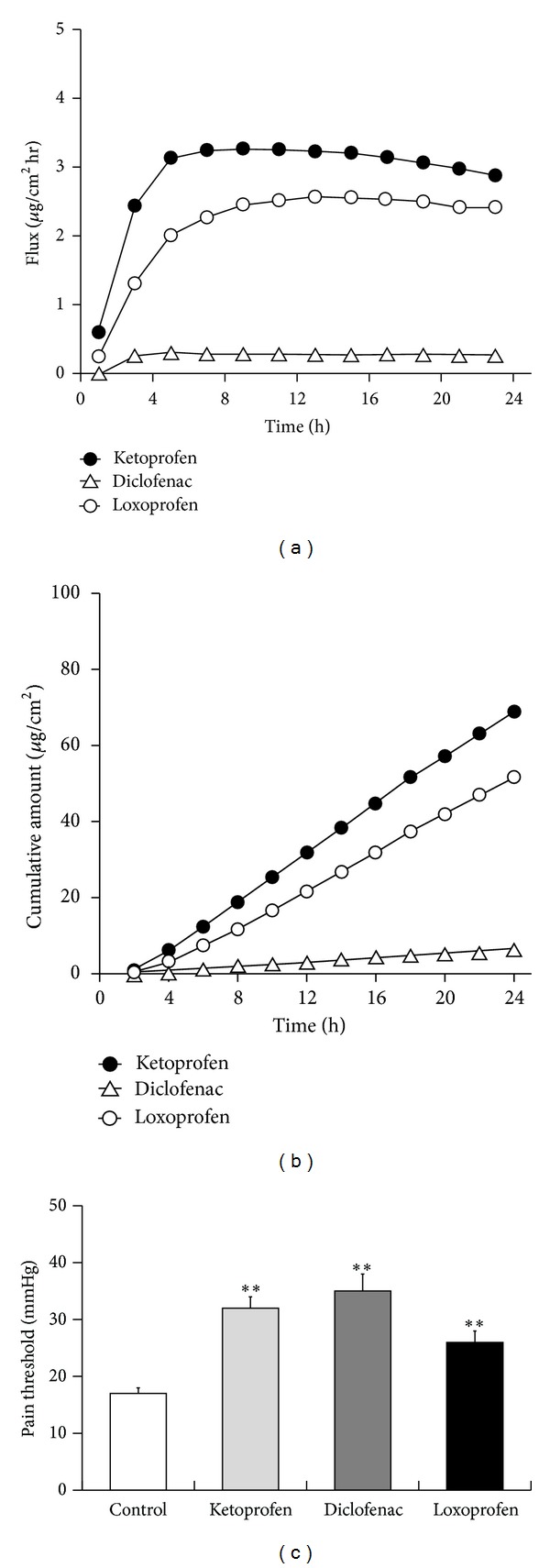
Advantages of ketoprofen as a topical NSAID. Cutaneous permeation profiles of multiple NSAIDs. The concentration of the test drug measured by liquid chromatography (a) and the cumulative amount of the drug calculated from the data shown in (a), (b). *N* = 5 in each condition. Hind paw hyperalgesia with direct NSAID injection in the affected site (c). Each column represents the mean ± SE of 4-5 animals. Asterisks show a significant difference from control, **P < 0.01.

The superiority of ketoprofen to other NSAIDs in terms of pain relief can be explained by 2 possible mechanisms. The first is its potentially higher permeation into the skin and the second is its potentially greater inhibitory activity on cyclooxygenase-2 (COX-2). To test the role of these mechanisms, an *in vitro* permeation study was conducted using hairless mouse skin. We found that the concentration of ketoprofen was the highest of all the NSAIDs tested at each time point, resulting in the highest cumulative amount (Figures 5(a) and 5(b)). Next, to compare the COX-2 inhibition of each NSAID, identical concentrations of each agent together with yeast were directly injected into the hind paw, and pain threshold was then examined. As shown in Figure 5(c), all 3 tested NSAIDs increased the pain threshold, and the effect of ketoprofen was not superior to that of the other 2 agents.

## 4. Discussion

In this study, we showed that STI is a sensitive parameter for the measurement of the therapeutic effects of analgesic agents, and for the first time, demonstrated the efficacy of topical NSAID treatments in an experimental animal model of acute inflammation. The carrageenan-induced inflammation model is one of several universal acute inflammation models [15]. However, as shown in Figures 2 and 3, neither histological analysis nor swelling measurement was sensitive enough to detect the therapeutic efficacy of topical NSAIDs. The yeast-induced inflammation model is also used to detect changes in pain threshold [14], although this approach was also unable to demonstrate the efficacy of topical NSAID treatment. Moreover, pain threshold measurement has the potential to introduce a subjective bias depending on the experimenter. In contrast, our gait analysis system can quantitatively detect the therapeutic effects of topical analgesic agents, suggesting the relative advantage of this approach for drug evaluation (Figures 2(a), 4(a), and 4(b)).

Superior effects of topical ketoprofen were observed in walking function (swing time index, Figure 2(a)) and relief in inflammatory edema (swelling rate, Figure 2(b)), when compared to other NSAIDs used in this study. Pain threshold was significantly improved by all tested NSAIDs, and ketoprofen exerted the most potent effect (Figure 2(c)). The superiority of ketoprofen may be due to its high permeability as shown in Figure 5. These results indicate ketoprofen as effective topical NSAIDs for patients with arthritis. Moreover, gait analysis can be applied to evaluate efficacy of drugs on walking function comprehensively, because it measures a physical response that directly correlates with local pain and inflammation [14, 15].

According to the data in Figure 4, pretreatment NSAID administration was superior to posttreatment NSAID administration, and the combination treatment was more effective than the monotherapy. In addition, STI improvement was more marked as the patch area increased (Figure 4(a)). This suggests that pain relief in clinical settings would be enhanced by the application of NSAIDs in as large an area as possible before walking or engaging in activities that are likely to cause pain. NSAIDs are widely used in first-line therapy for arthritis with pain [17]. Acetaminophen and weak opioids are sometimes used in combination with NSAIDs for arthritis with resistant pain. However, controlling chronic pain with oral administration of these drugs still has difficulties, as well as serious adverse effects [18, 19]. Moreover, transudation of the orally administered NSAIDs in the synovial fluid of the patients with arthritis has been reported to be lower than that in circulation, indicating high dose administration is needed for relief pain in arthritis [20]. Thus, topical application of NSAIDs may be more useful than oral administration not only to circumvent the adverse effects but also to exert maximum effects.

In conclusion, we demonstrated the advantage of gait analysis for the detection of pain relief in an *in vivo* experimental model of acute inflammation. Furthermore, we showed the potential clinical utility of topical ketoprofen for the treatment of pain. We believe that these results will provide a novel strategy for drug screening.
